# 2-D Joint Sparse Reconstruction and Micro-Motion Parameter Estimation for Ballistic Target Based on Compressive Sensing

**DOI:** 10.3390/s20164382

**Published:** 2020-08-05

**Authors:** Jiaqi Wei, Shuai Shao, Lei Zhang, Hongwei Liu

**Affiliations:** 1 National Lab of Radar Signal Processing, Xidian University, Xi’an 710071, China; weijiaqi_0831@126.com (J.W.); hwliu@xidian.edu.cn (H.L.); 2 School of Electronics and Communication Engineering, Sun Yat-Sen University, Guangzhou 510275, China; zhanglei57@mail.sysu.edu.cn

**Keywords:** ballistic target, micro-motion, sparse frequency band (SFB) signal, two-dimension (2-D) joint sparse reconstruction, two-dimension (2-D) joint parameter estimation

## Abstract

The sparse frequency band (SFB) signal presents a serious challenge to traditional wideband micro-motion curve extraction algorithms. This paper proposes a novel two-dimension (2-D) joint sparse reconstruction and micro-motion parameter estimation (2D-JSR-MPE) algorithm based on compressive sensing (CS) theory. In this technique, the 2D-JSR signal model and the micro-motion parameter dictionary are established based on the segmented SFB echo signal, in which the idea of piecewise effectively reduces the model complexity of ballistic target. With the accommodation of the CS theory, the 2D-JSR-MPE of the echo signal is transformed into solving a sparsity-driven optimization problem. Via an improved orthogonal matching pursuit (OMP) algorithm, the high-resolution range profiles (HRRP) can be reconstructed accurately, and the precise micro-motion curves can be simultaneously extracted on phase accuracy. The employment of 2-D joint processing can effectively avoid the interference of the sparse reconstruction error caused by cascaded operation in the subsequent micro-motion parameter estimation. The proposed algorithm benefits from the anti-jamming characteristic of the SFB signal and 2-D joint processing, thus remarkably enhancing its accuracy, robustness and practicality. Extensive experimental results are provided to verify the effectiveness and robustness of the proposed algorithm.

## 1. Introduction

The ballistic missile has become the leading weapon in modern wars. Missiles are often accompanied by decoys in the middle of the flight trajectory, and the micro-motion characteristics of warheads and decoys are obviously variant depending on their mass distribution. This makes estimating the target’s micro-motion parameters an important technique for target recognition in the missile defense system [1,2,3,4]. At present, methods such as phase derived ranging (PDR) technique are commonly used to extract micro-motion features of the ballistic target by processing wideband echo signals. By recourse to the phase information of range profile of the wideband echo signal, this type of approach can accurately depict the motion state of the scattering center and extract the micro-motion feature, thereby making the ranging accuracy reach the level of half a wavelength [5,6]. However, the traditional PDR algorithm above is based on a high-resolution range profile (HRRP) and does not use the 2-D coherent accumulation gain of echo signal, so it is susceptible to noise. In addition, the wideband radar is prone to environmental interference, which undermines its stability. The wideband signal requires the receiver to have the corresponding instantaneous bandwidth, posing a severe challenge to the design of the radar system and limiting its practicality in reality. In order to solve these problems, a sparse frequency band (SFB) signal has been developed, including a sparse orthogonal frequency division multiplexing (OFDM) signal and a sparse stepped-frequency signal [7,8]. Only transmitting part of the full frequency band (FFB), the SFB signal can effectively avoid certain interference frequency bands and reduce the data sampling amount, thereby improving the anti-jamming ability of the radar system and decreasing the operation burden of the radar hardware. However, the SFB signal severely challenges the traditional micro-motion curve extraction algorithms. Hence, it is of great significance to study the micro-motion extraction in SFB.

For the SFB signal, the reconstruction of HRRP is essential. The existing sparse reconstruction methods can be classified into four main categories. The first is nonlinear filtering represented by sequence CLEAN (S-CLEAN) [9,10]. It suppresses the high grating and side lobes of the pulse response function by remaining the peak value of the main lobe. The second is a bandwidth extrapolation (BWE) algorithm, such as the Burg algorithm [11,12]. This kind of method usually uses the linear prediction model to fit observation data, then adopts the spectrum estimation method to estimate the model parameters, and improves resolution through aperture extrapolation. The third is the parametric spectrum estimation technique, including the RELAX algorithm [13,14]. This type of algorithm establishes a parametric model for the echo signal, and precisely estimates the positions and amplitudes of the scattering centers through spectrum estimation so as to achieve high-resolution imaging. However, the above algorithms are all easily affected by noise and model errors, so the sparse reconstruction algorithm based on compressive sensing (CS) theory has been proposed in recent years. This kind of algorithm fulfills sparse data reconstruction by solving an $l_{1}$-norm optimization problem, and thus is highly robust against noise [15,16]. When it comes to the micro-motion target, the 2-D coupling problem exists in the echo signal duo to micro-motion. Thus, if the 2-D joint micro-motion parameter estimation can be achieved simultaneously with sparse reconstruction, higher accuracy in estimation and stronger robustness can be guaranteed. However, by implementing sparse reconstruction in a single dimension, the above four types of methods fail to make the most of the 2-D coupling information and the 2-D coherent accumulation gain of the echo signal, which adversely affects the accuracy of signal reconstruction and parameter estimation as well as the robustness against noise.

Inspired by the above problems, this paper presents a 2-D joint sparse reconstruction and micro-motion parameter estimation (2D-JSR-MPE) algorithm aimed at the SFB signal. In this technique, a novel 2-D joint sparse reconstruction signal model combined with a segmented micro-motion characteristic parameter dictionary is established, in which the piecewise processing effectively reduces the model complexity of ballistic target. Based on this, a 2D-JSR-MPE optimization function is developed by means of the CS sparse representation, transforming the sparse reconstruction and parameter estimation into solving a sparsity-driven optimization problem with $l_{1}$-norm. With the accommodation of the improved orthogonal matching pursuit (OMP) algorithm [17], micro-motion parameter estimation and sparse reconstruction can be simultaneously realized, thereby the high-precision HRRP and micro-motion curve can be obtained. Two-dimensional joint processing makes it possible to prevent the interference of the sparse reconstruction error in micro-motion parameter estimation, thus effectively avoiding the adverse effect of error transmission. The proposed algorithm estimates the micro-motion parameters by processing the phase information, so the estimation accuracy can reach the level of half a wavelength. Moreover, since the 2-D joint processing makes the most of the 2-D coupling information and 2-D coherent accumulation gain of the echo signal, and the SFB signal performs well in anti-jamming with low computational complexity, the proposed algorithm has the advantages of high accuracy, strong robustness, good practicality and low operational burden. Abundant experimental results corroborate the superiority of the proposed algorithm.

This paper is organized as follows. Section 2 introduces the 2D-JSR-MPE signal model of the ballistic target. Section 3 explicates the 2D-JSR-MPE algorithm. Section 4 provides the experimental results to illustrate the effectiveness of the proposed algorithm. The paper ends with a brief conclusion in Section 5.

## 2. Signal Model

### 2.1. The Micro-Motion Model of Ballistic Target

**Figure 1:**
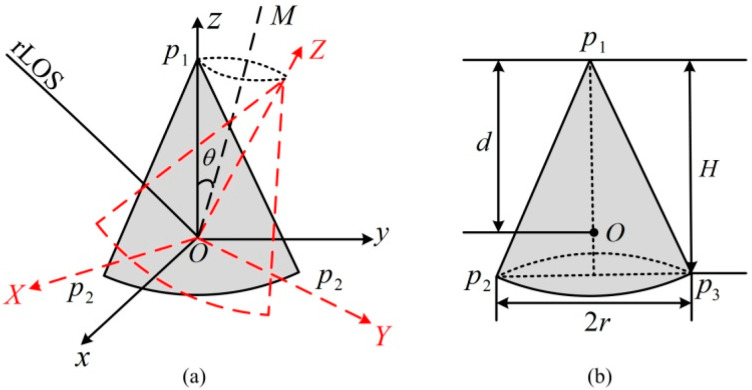
Ballistic target model: (**a**) Micro-motion model; (**b**) Geometry model.

The micro-motion model of ballistic target is shown in Figure 1a. (x,y,z) is the radar coordinate system and (X,Y,Z) are the target body coordinate system. RLOS is the radar line of sight, and the elevation angle and the azimuth angle of RLOS are α and β, respectively. The geometry model of ballistic target is shown in Figure 1b. H denotes the height of the cone, r refers to the radius of the bottom, and d represents the distance between the mass center and the top of the cone. For the ballistic cone target model established in this paper, there are three equivalent scattering centers on the target [2], namely $p_{1}$, $p_{2}$ and $p_{3}$ in Figure 1b. When moving in precession, the target makes a spin motion on its axis of symmetry at an angular velocity of $\omega_{s}$, and makes a conic motion on the conic axis OM at an angular velocity of $\omega_{c}$. The precession angle is θ. When the target moves in nutation, on the basis of precession, the cone axis of the target oscillates in accordance with the sine wave within a certain range. The oscillation angle is $\kappa \left(t_{m}\right)=\delta \sin\left(\omega_{v}t_{m}\right)$; δ and $\omega_{v}$ stand for the maximum amplitude and the angular velocity of oscillation, respectively. Given that the ballistic cone target studied in this paper is axisymmetric, its spin motion does not affect the radar echo of the target and is therefore not taken into consideration.

In this paper, it is assumed that the translational motion of the target has been compensated, then the instantaneous radial distance between the kth scattering center and the radar can be calculated by the projection from the kth scattering center to RLOS

(1) $$r_{k}(t_{m})=Y_{k}\sin\gamma \left(t_{m}\right)+Z_{k}\cos\gamma \left(t_{m}\right)+R_{0}$$

where $R_{0}$ represents the distance from the mass center of target to the radar; $\gamma \left(t_{m}\right)$ denotes the angle between the symmetry axis of the target and RLOS at $t_{m}$. When the target moves in precession, the cosine of the angle between the symmetry axis of the target and RLOS can be expressed as (2)

(2) $$\cos\gamma (t_{m})=\cos\theta \cos(\theta +\alpha )+\sin\theta \sin(\theta +\alpha )\cos(\omega_{c}t_{m}+\varphi )$$

where φ denotes initial phase. When the target moves in nutation, the cosine of the angle between the symmetry axis of the target and RLOS can be expressed as (3)

(3) $$\begin{array}{ll} \cos\gamma \left(t_{m}\right) & =\cos\theta \left[\cos\left(\theta +\alpha \right)\cos\left[\kappa \left(t_{m}\right)\right]-\sin\left(\alpha +3\theta \right)\sin\left[\kappa \left(t_{m}\right)\right]\right]\\ & +\sin\theta \cos\left(\omega_{c}t_{m}\right)\left[\cos\left(\alpha +3\theta \right)\sin\left[\kappa \left(t_{m}\right)\right]-\sin\left(\theta +\alpha \right)\cos\left[\kappa \left(t_{m}\right)\right]\right] \end{array}$$

Assigning (2) or (3) to (1), the theoretical expression of the instantaneous distance between each scattering center and the radar can be given by (4)

(4) $$\{\begin{array}{l} r_{k}\left(t_{m}\right)=R_{0}+R_{k}\left(t_{m}\right),k=1,2,3\\ R_{1}\left(t_{m}\right)=-d\cos\gamma \left(t_{m}\right)\\ R_{2}\left(t_{m}\right)=\left(H-d\right)\cos\gamma \left(t_{m}\right)-r\sin\gamma \left(t_{m}\right)\\ R_{3}\left(t_{m}\right)=\left(H-d\right)\cos\gamma \left(t_{m}\right)+r\sin\gamma \left(t_{m}\right) \end{array}$$

where $R_{k}\left(t_{m}\right)$ represents the instantaneous micro distance of each scattering center. It can be seen that the instantaneous micro distance of each scattering center changes with time in a form approximating to the sine wave and includes the target’s motion and geometry parameters.

### 2.2. D-JSR-MPE Signal Model

It is assumed that the radar transmits a linear frequency modulated (LFM) signal. After preprocessing such as range compression and de-modulation to the baseband, the received signal of the kth scattering center can be given by (5) [18]

(5) $$S^{k}\left(f_{r},t_{m}\right)=\sigma_{k}\cdot \exp\left[-j4\pi \frac{\left(f_{c}+f_{r}\right)}{c}\cdot R_{k}\left(t_{m}\right)\right]$$

where $\sigma_{k}$ represents the scattering coefficient of the kth scattering center; c denotes the light velocity. From (4), it can be seen that the instantaneous micro distance of each scattering center can be expressed as a sum of the multi-order Sine functions, but it is complex to cope with such forms in practice. Thus, underpinned by the idea of piecewise [19], in a short period of time, using a second-order polynomial model to approximate the instantaneous micro distance of scattering points can reduce the complexity of the model effectively. Moreover, because of the short dwell time, the second-order polynomial is accurate enough to fit the instantaneous micro-motion trajectory of the ballistic target, which is conducive to the subsequent reconstruction of the micro-motion curve. In this paper, the echo signal is segmented evenly, and the approximate expression of the instantaneous micro distance of the jth segment of the kth scattering point can be written as (6)

(6) $$R_{j}^{k}\left(t_{m}\right)=a_{j}^{k}+b_{j}^{k}t_{m}+c_{j}^{k}t_{m}^{2}$$

where $a_{j}^{k}$, $b_{j}^{k}$ and $c_{j}^{k}$ denote micro-motion parameters. By estimating them accurately, the micro-motion curve can be reconstructed. Assigning (6) to (5), the jth segmented echo signal can be given by (7)

(7) $$S_{j}^{k}\left(f_{r},t_{m}\right)=\sigma_{k}\cdot \exp\left[-j4\pi \frac{\left(f_{c}+f_{r}\right)}{c}\cdot \left(a_{j}^{k}+b_{j}^{k}t_{m}+c_{j}^{k}t_{m}^{2}\right)\right]$$

After sampling processing, the discrete form of (7) can be expressed as (8)

(8) $$S_{j}^{k}\left(n,m\right)=\sigma_{k}\cdot \exp\left[-j4\pi \frac{\left(f_{c}+n\Delta f\right)}{c}\cdot \left(a_{j}^{k}+b_{j}^{k}\left(m\Delta t\right)+c_{j}^{k}{\left(m\Delta t\right)}^{2}\right)\right]$$

where n refers to the range frequency bin index, and m the azimuth slow time index. The ranges of n and m are as follows: n∈[−N/2 +1:N/2 ] and m∈[−M/2 +1:M/2 ], where N refers to the total number of the range frequency bin of FFB signal, and M stands for the number of pulses contained in the jth segmented echo signal. Δf corresponds to the range frequency sampling interval, and Δt the slow time sampling interval.

**Figure 2:**
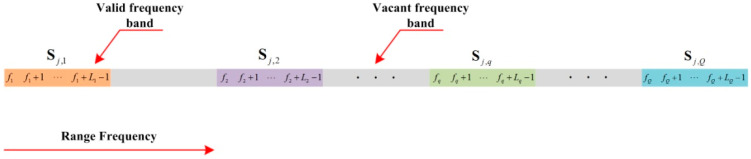
The geometry model of SFB signal.

Equation (8) is the expression corresponding to the FFB signal. When the range frequency band is sparse, the geometry model of the SFB signal is shown in Figure 2. It is assumed that the SFB signal consists of Q sparse subbands, in which the qth subband is $L_{q}$ in length, running from $f_{q}$ to $f_{q}+L_{q}-1$, and $f_{q}$ denotes the initial range frequency bin index of the qth subband. It is assumed that the qth subband signal can be represented by the matrix $S_{j,q}^{k}$ which is $L_{q}\times M$ in size. Then, (8) can be expressed in the form of matrix, as shown in (9)

(9) $$S_{j}^{k}={\left[\begin{array}{ccccc} S_{j,1}^{k} & \cdots & S_{j,q}^{k} & \cdots & S_{j,Q}^{k} \end{array}\right]}_{\bar{L}\times M}$$

where $\bar{L}=\sum_{q=1}^{Q}L_{q}$. Assuming that there are a total of K scattering centers in the target, the SBF echo signal of the target can be expressed as (10)

(10) $$S_{j}={\left[\begin{array}{ccccc} S_{j,1} & \cdots & S_{j,q} & \cdots & S_{j,Q} \end{array}\right]}_{\bar{L}\times M}$$

where $S_{j,q}=\sum_{k=1}^{K}S_{j,q}^{k}$. Considering the inevitable noise and combined with (8) and (10), $S_{j}$ can be re-expressed as the following matrix form

(11) $$S_{j}=D_{j}⊙F_{r}\cdot Y_{j}^{T}\cdot F_{a}^{T}+N_{j}$$

where ⊙ represents the Hadamard product and $D_{j}\in {\mathbb{C}}^{\bar{L}\times M}$ denotes the micro-motion parameter matrix as (12)

(12) $$D_{j}={\left[\begin{array}{cccc} \exp\left(-j\frac{4\pi \left(\Delta f+f_{c}\right)}{c}P_{j}\left(\Delta t\right)\right) & \exp\left(-j\frac{4\pi \left(\Delta f+f_{c}\right)}{c}P_{j}\left(2\Delta t\right)\right) & \cdots & \exp\left(-j\frac{4\pi \left(\Delta f+f_{c}\right)}{c}P_{j}\left(M\Delta t\right)\right)\\ \vdots & \vdots & \ddots & \vdots \\ \exp\left(-j\frac{4\pi \left(L_{1}\Delta f+f_{c}\right)}{c}P_{j}\left(\Delta t\right)\right) & \exp\left(-j\frac{4\pi \left(L_{1}\Delta f+f_{c}\right)}{c}P_{j}\left(2\Delta t\right)\right) & \cdots & \exp\left(-j\frac{4\pi \left(L_{1}\Delta f+f_{c}\right)}{c}P_{j}\left(M\Delta t\right)\right)\\ \vdots & \vdots & \ddots & \vdots \\ \exp\left(-j\frac{4\pi \left(\bar{L}\Delta f+f_{c}\right)}{c}P_{j}\left(\Delta t\right)\right) & \exp\left(-j\frac{4\pi \left(\bar{L}\Delta f+f_{c}\right)}{c}P_{j}\left(2\Delta t\right)\right) & \cdots & \exp\left(-j\frac{4\pi \left(\bar{L}\Delta f+f_{c}\right)}{c}P_{j}\left(M\Delta t\right)\right) \end{array}\right]}_{\bar{L}\times M}$$

where $P_{j}\left(m\Delta t\right)=b_{j}\left(m\Delta t\right)+c_{j}{\left(m\Delta t\right)}^{2}$, m=1,2,⋯M, the ranges of $b_{j}$ and $c_{j}$ are as follows: $b_{j}\in \left[-\eta /2\;:\eta /2\;\right]$ and $c_{j}\in \left[-\mu /2\;:\mu /2\;\right]$, in which η and μ should be large enough to ensure that $b_{j}$ and $c_{j}$ can include the center frequencies and chirp rates of all sub-segments echoes. $F_{r}={\left[F_{r0};\cdots ;F_{rq};\cdots ;F_{r\left(Q-1\right)}\right]}_{\bar{L}\times N}$ represents the partial Fourier dictionary of range dimension, $F_{rq}={\left[f_{rq}^{f_{q}};\cdots ;f_{rq}^{f_{q}+L_{q}-1}\right]}_{L_{q}\times N}$, where $f_{rq}^{x}=\left[1,\omega_{r}^{x},\cdots ,\omega_{r}^{\left(N-1\right)x}\right]$, $x=f_{q},f_{q}+1,\cdots ,f_{q}+L_{q}-1$, $\omega_{r}=\exp\left[-j2\pi /N\;\right]$. $F_{a}\in {\mathbb{C}}^{M\times M}$ denotes the standard Fourier dictionary of azimuth dimension, whose detailed form is not given here. $Y_{j}\in {\mathbb{C}}^{M\times N}$ corresponds to the 2-D full-resolution ISAR image and $N_{j}\in {\mathbb{C}}^{\bar{L}\times M}$ the additive complex Gaussian white noise matrix.

Equation (11) is the 2D-JSR-MPE signal model proposed in this paper. In the next section, we will explicate the HRRP reconstruction and micro-motion curve estimation of scattering centers based on the CS theory.

## 3. D-JSR-MPE Algorithm

### 3.1. The Principle of 2D-JSR-MPE Based on CS

The CS theory points out that by analyzing the sparsity of the echo signal, constructing the measurement matrix, and using the existing optimization algorithms, the sparse signal can be reconstructed with a little data sampling amount. Combined with the derivation in the previous section, in order to facilitate the construction and solution of the optimization function, the vectorization operation is carried out for (11)

(13) $$vec\left[S_{j}\right]=vec\left[D_{j}⊙F_{r}\cdot Y_{j}^{T}\cdot F_{a}^{T}\right]+vec\left[N_{j}\right]$$

where vec[·] refers to the vectorization operation. For clarity, we mark $s_{j}=vec\left[S_{j}\right]\in {\mathbb{C}}^{\left(\bar{L}\cdot M\right)\times 1}$, $y_{j}=vec\left[Y_{j}^{T}\right]\in {\mathbb{C}}^{\left(N\cdot M\right)\times 1}$, and $n_{j}=vec\left[N_{j}\right]\in {\mathbb{C}}^{\left(\bar{L}\cdot M\right)\times 1}$. Based on this, (13) can be expressed as (14) according to the relationship between vectorization operation and Kronecker product [20,21,22]

(14) $$s_{j}=G_{j}\cdot \left(F_{a}⊗F_{r}\right)\cdot y_{j}+n_{j}=G_{j}\cdot W\cdot y_{j}+n_{j}$$

where ⊗ represents Kronecker product; $W\in {\mathbb{C}}^{\left(\bar{L}\cdot M\right)\times \left(N\cdot M\right)}$ denotes the 2-D joint sparse reconstruction dictionary, and $W=F_{a}⊗F_{r}$. In particular, $G_{j}\in {\mathbb{C}}^{\left(\bar{L}\cdot M\right)\times \left(\bar{L}\cdot M\right)}$ is a diagonal matrix composed of all elements in $D_{j}$ as the diagonal elements. Based on the CS theory and (14), we can establish a 2D-JSR-MPE optimization function as (15)

(15) $$\min{\left\|y_{j}\right\|}_{1},st.{\left\|s_{j}-G_{j}\cdot W\cdot y_{j}\right\|}_{2}<\varepsilon_{j}$$

where $\varepsilon_{j}$ represents the noise threshold; ${\left\|\cdot \right\|}_{i}$ denotes the i-norm of vector. According to the CS theory, 2D-JSR-MPE can be realized by solving the $l_{1}$-norm optimization problem shown in (15). Therefore, in the next subsection, we will elucidate an algorithm which can quickly and accurately address the optimization problem shown in (15).

### 3.2. 2D-JSR-MPE Based on the Improved OMP Algorithm

Due to the large size of $G_{j}\cdot W$, it is difficult for common algorithms to solve (15). In this subsection, via fast Fourier transform (FFT), the improved OMP algorithm is employed to jointly realize HRRP reconstruction and micro-motion curve estimation. The improved OMP algorithm mainly includes the following four steps:

(1) Micro-motion curve parameters estimation. Firstly, the micro-motion curve parameters of the first scattering center in $s_{j}$ are estimated by (16)
  (16) $$\langle a_{j}^{1},b_{j}^{1},c_{j}^{1}\rangle =\max\left|{\left(H_{j}\cdot W\right)}^{H}s_{j}\right|$$
  where $H_{j}=A_{j}⊙G_{j}$, and $A_{j}\in {\mathbb{C}}^{\left(\bar{L}\cdot M\right)\times \left(\bar{L}\cdot M\right)}$ is a diagonal matrix composed of all elements in $a_{j}$ as diagonal elements as (17)
  (17) $$a_{j}={\left[\begin{array}{cccc} \exp\left(-j\frac{4\pi \left(\Delta f+f_{c}\right)}{c}a_{j}\right) & \exp\left(-j\frac{4\pi \left(\Delta f+f_{c}\right)}{c}a_{j}\right) & \cdots & \exp\left(-j\frac{4\pi \left(\Delta f+f_{c}\right)}{c}a_{j}\right)\\ \vdots & \vdots & \ddots & \vdots \\ \exp\left(-j\frac{4\pi \left(L_{1}\Delta f+f_{c}\right)}{c}a_{j}\right) & \exp\left(-j\frac{4\pi \left(L_{1}\Delta f+f_{c}\right)}{c}a_{j}\right) & \cdots & \exp\left(-j\frac{4\pi \left(L_{1}\Delta f+f_{c}\right)}{c}a_{j}\right)\\ \vdots & \vdots & \ddots & \vdots \\ \exp\left(-j\frac{4\pi \left(\bar{L}\Delta f+f_{c}\right)}{c}a_{j}\right) & \exp\left(-j\frac{4\pi \left(\bar{L}\Delta f+f_{c}\right)}{c}a_{j}\right) & \cdots & \exp\left(-j\frac{4\pi \left(\bar{L}\Delta f+f_{c}\right)}{c}a_{j}\right) \end{array}\right]}_{\bar{L}\times M}$$

For solving (16), the traditional method needs to assign all values of $\langle a_{j}^{1},b_{j}^{1},c_{j}^{1}\rangle$ to $H_{j}$, respectively, the computational complexity of each iteration is $O\left(NM^{2}\bar{L}\right)$, which greatly increases the computational amount. In this paper, FFT is introduced into the process of solving $\langle a_{j}^{1},b_{j}^{1},c_{j}^{1}\rangle$

(18) $$U=FFT2\left\{S_{j}⊙D_{j}\right\}$$

where FFT2{·} represents the 2-D fast Fourier transform (2D-FFT). Based on (18), $\langle {\hat{a}}_{j}^{1},{\hat{b}}_{j}^{1},{\hat{c}}_{j}^{1}\rangle$, the optimal estimation of $\langle a_{j}^{1},b_{j}^{1},c_{j}^{1}\rangle$, can be determined by searching the maximum value of U. Then, the basis matrix corresponding to SFB $\Phi_{j}=\left[H_{j,\langle {\hat{a}}_{j}^{1},{\hat{b}}_{j}^{1},{\hat{c}}_{j}^{1}\rangle }\right]$ can be constructed by $\langle {\hat{a}}_{j}^{1},{\hat{b}}_{j}^{1},{\hat{c}}_{j}^{1}\rangle$, and the homologous basis matrix with respect to FFB established as $\Phi_{j}^{FFB}$.

(2) Scattering center estimation. After obtaining $\Phi_{j}$, the weighted least square estimation (WLSE) method is adopted to estimate the principal component of the scattering centers in $y_{j}$, and the corresponding scattering coefficient is ${\hat{\Psi }}_{j}=\left[{\hat{\Psi }}_{j}^{1}\right]={\left(\Phi_{j}^{H}\Phi_{j}\right)}^{-1}\Phi_{j}^{H}S_{j}$. On this basis, we can reconstruct the corresponding SFB observation data ${\hat{S}}_{j}=\Phi_{j}{\hat{\Psi }}_{j}$.

(3) Residual signal estimation. First, the residual SFB signal ${\bar{S}}_{j}$ is calculated by subtracting ${\hat{S}}_{j}$ from $S_{j}$, i.e., ${\bar{S}}_{j}=S_{j}-{\hat{S}}_{j}$. Then, according to step 1 and step 2, the parameter estimation and scattering center extraction of the principle component signal in ${\bar{S}}_{j}$ are carried out. The estimated micro-motion curve parameters are $\langle {\hat{a}}_{j}^{2},{\hat{b}}_{j}^{2},{\hat{c}}_{j}^{2}\rangle$. In this case, $\langle {\hat{a}}_{j}^{2},{\hat{b}}_{j}^{2},{\hat{c}}_{j}^{2}\rangle$ are used to update the basis matrix to $\Phi_{j}=\left[H_{j,\langle {\hat{a}}_{j}^{1},{\hat{b}}_{j}^{1},{\hat{c}}_{j}^{1}\rangle },H_{j,\langle {\hat{a}}_{j}^{2},{\hat{b}}_{j}^{2},{\hat{c}}_{j}^{2}\rangle }\right]$, and the scattering coefficients are estimated as ${\hat{\Psi }}_{j}=\left[{\hat{\Psi }}_{j}^{1};{\hat{\Psi }}_{j}^{2}\right]={\left(\Phi_{j}^{H}\Phi_{j}\right)}^{-1}\Phi_{j}^{H}S_{j}$. It should be noted that the scattering coefficient of the principal component signal estimated in the first time and $\Phi_{j}^{FFB}$ are also updated here.

(4) Iterative operation. Repeat Step 2 and Step 3 until ${\left\|{\bar{S}}_{j}\right\|}_{2}<\varepsilon_{j}$ is met.

Through the above processing, the micro-motion curve of the kth scattering center of the jth segment can be reconstructed by (19)

(19) $${\hat{R}}_{j}^{k}\left(t_{m}\right)={\hat{a}}_{{}_{j}}^{k}+{\hat{b}}_{{}_{j}}^{k}t_{m}+{\hat{c}}_{{}_{j}}^{k}t_{m}^{2}$$

Moreover, the reconstructed HRRP of the jth segment can be obtained using range inverse FFT (IFFT) for the reconstructed FFB signal ${\hat{S}}_{j}^{FFB}=\Phi_{j}^{FFB}{\hat{\Psi }}_{j}$. The final reconstructed HRRPs and micro-motion curves can be obtained by splicing and integrating the processing results of each subsegment. Through 2-D joint processing, sparse reconstruction and micro-motion parameter estimation are carried out simultaneously, which avoids the cascaded error transfer resulting from “reconstruction first and then estimation”, thus improving the accuracy and robustness of parameter estimation. Since the proposed algorithm taps the phase information to estimate the micro-motion parameters, the estimation accuracy can reach the level of half a wavelength, rendering the proposed algorithm highly accurate in terms of parameter estimation.

The computational complexity of the proposed algorithm mainly arises from the FFT operation in Step 1 and the matrix inversion operation in Step 2. The computational complexity of the matrix inversion operation in Step 2 is $O\left(\sum_{k=1}^{K}k^{3}\right)$, which can be neglected since there are only a few equivalent scattering centers for the ballistic target studied in this paper. Therefore, it is Step 1 that mainly determines the complexity of the proposed algorithm, which can be expressed by the number of complex multiplications [23]. It can be seen from (18) that 2D-FFT is employed to estimate the micro-motion parameters of each subsegment. The computational complexity of 2D-FFT is $O\left(M\cdot N\log_{2}\left(M\cdot N\right)\right)$, and the search ranges of $b_{j}$ and $c_{j}$ are η and μ, respectively, so the computational complexity corresponding to each subsegment is $O\left(\eta \cdot \mu \cdot M\cdot N\log_{2}\left(M\cdot N\right)\right)$. Given that the echo signal is divided into J segments, the total computational complexity of the proposed algorithm is $O\left(J\cdot \eta \cdot \mu \cdot M\cdot N\log_{2}\left(M\cdot N\right)\right)$. Through FFT and Hadamard product, the computational complexity of each iteration of the proposed algorithm $O\left(M\cdot N\log_{2}\left(M\cdot N\right)\right)$ is much smaller than that of direct matrix operation $O\left(NM^{2}\bar{L}\right)$, so the efficiency of the 2-D joint processing algorithm can be effectively improved by fast operation.

**Figure 3:**
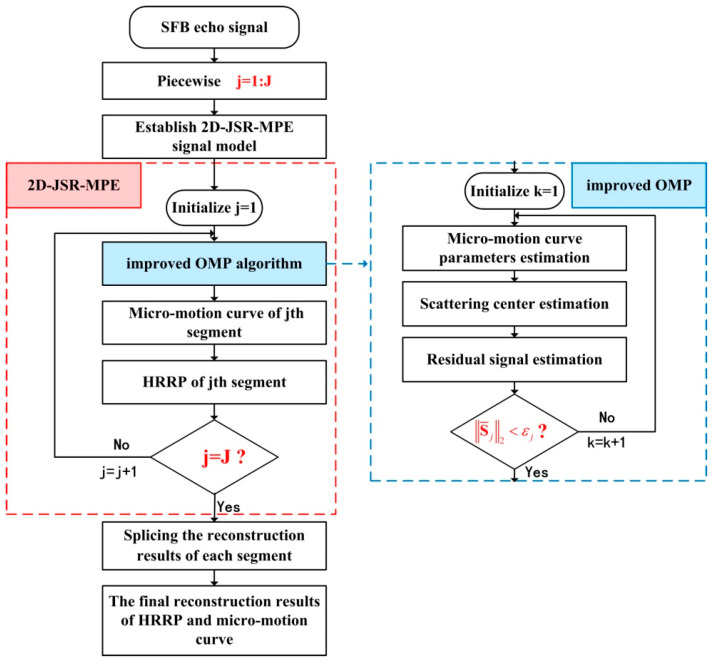
Flowchart of 2D-JSR-MPE.

In order to describe the proposed algorithm clearly, a flowchart is given in Figure 3.

## 4. Experiments and Analyses

The geometry model of the ballistic target shown in Figure 1b is adopted in this simulation experiment, and the main simulated target and radar system parameters are shown in Table 1 and Table 2, respectively. In this experiment, three comparison algorithms are employed to illustrate the advantages of the proposed algorithm. The ‘zero-padding FFT (ZP-FFT)’ method [8] carries out a zero padding operation on the part of the vacant frequency band, and obtains the range profiles of the scattering centers through range IFFT. For the ‘S-CLEAN’ method, the HRRPs of the scattering centers are reconstructed by the S-CLEAN algorithm [10], and then the micro-motion curve is extracted by the traditional PDR algorithm. The ‘2-D cascaded processing algorithm (2D-CPA)’ uses the OMP algorithm [17] to reconstruct HRRP in the range dimension first, and then superimposes the target support area along the range bin. For the superimposed one-dimension (1-D) vector, the modified discrete chirp Fourier transform (MDCFT) [24] is utilized to estimate the parameters and reconstruct the micro-motion curve in the azimuth dimension. With respect to the proposed algorithm, for clarity, we name it as ‘2D-JSR-MPE’. It is worth noting that the HRRPs obtained by ZP-FFT have high gating and side lobes, so ZP-FFT is not applied to the subsequent estimation of micro-motion parameters in this experiment. In what follows, the effectiveness and superiority of the proposed algorithm are demonstrated by the experimental results obtained by the three comparison algorithms and the proposed algorithm at different signal to noise ratios (SNRs) and sparse rates (SRs).

**Figure 4:**
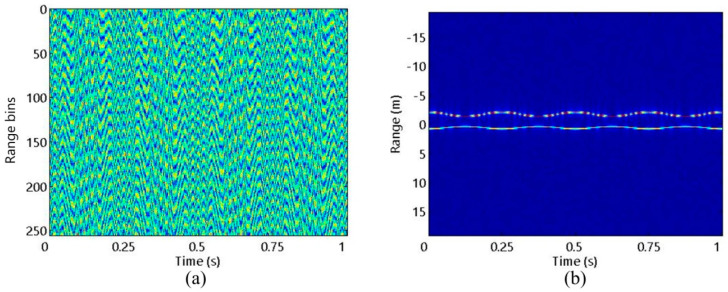
FFB situation: (**a**) Echo waveform; (**b**) HRRPs.

**Figure 5:**
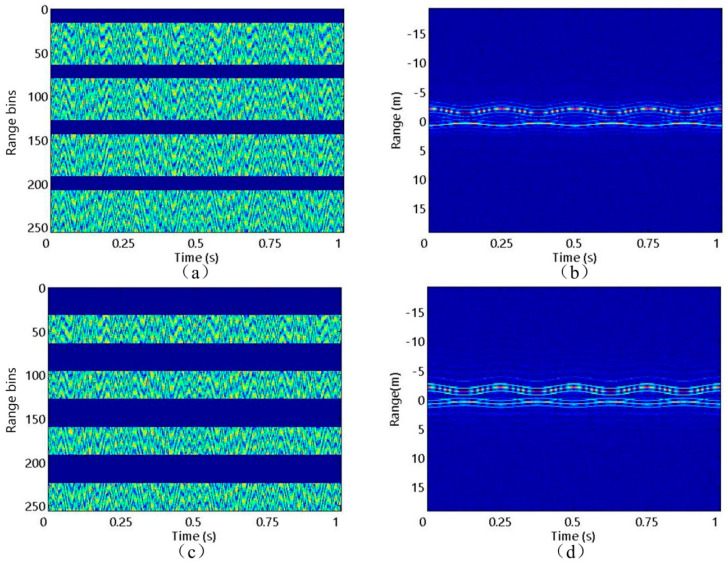
SFB situation. SR1: (**a**) Echo waveform; (**b**) HRRPs obtained by ZP-FFT. SR2: (**c**) Echo waveform; (**d**) HRRPs obtained by ZP-FFT.

First, the precession motion of the target is studied, and the experiment is carried out at SNR = 10 dB. Figure 4a shows the FFB echo waveform in range-frequency and azimuth-time domains at SNR = 10 dB with its corresponding HRRPs in Figure 4b. It can be seen that due to shielding effect, only one micro-motion curve corresponding to scattering centers $p_{2}$ or $p_{3}$ can be observed [25]. It is widely acknowledged that the sparse sampling pattern can be classified into two categories: gap missing sampling (GMS) and random missing sampling (RMS). They are only different in the sampling method, while identical in the subsequent experimental processing. The SFB signal (such as the sparse OFDM signal) is usually transmitted in the form of GMS. Therefore, in this experiment, we use GMS to illustrate the performance of the algorithms. In addition, two kinds of SRs are set, SR1 = 0.75 and SR2 = 0.5. The range dimension sampling points corresponding to SR1 and SR2 are three fourths and half as many as the FFB sampling points in Figure 4a, respectively. Figure 5 shows the SFB waveforms at SR1 and SR2 as well as the HRRPs obtained by ZP-FFT. As shown in Figure 5b,d, the HRRPs obtained by ZP-FFT have high gating and side lobes with low resolution, which seriously hinders the follow-up work.

**Figure 6:**
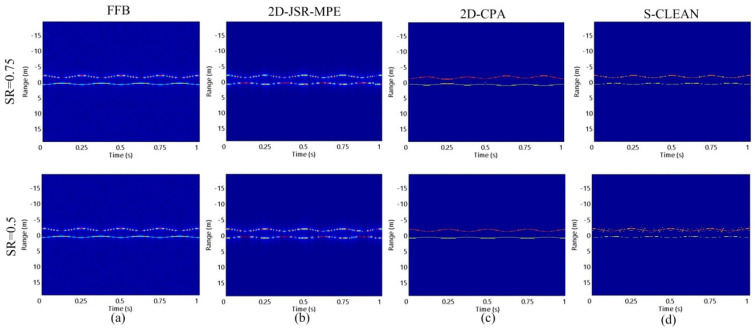
The reconstructed results of HRRP of the target with precession motion obtained by different algorithms with different SRs at SNR = 10 dB: (**a**) FFB situation; (**b**) 2D-JSR-MPE; (**c**) 2D-CPA; (**d**) S-CLEAN.

**Figure 7:**
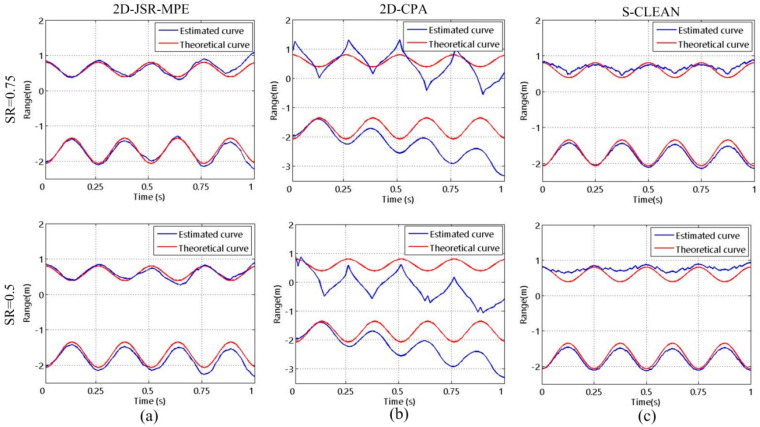
The reconstructed results of micro-motion curves of the target with precession motion obtained by different algorithms with different SRs at SNR = 10 dB: (**a**) 2D-JSR-MPE; (**b**) 2D-CPA; (**c**) S-CLEAN.

In the following experiment, we study the influence of the SR on the algorithms. When the target is in precession, the reconstructed results of HRRP obtained by 2D-JSR-MPE, 2D-CPA and S-CLEAN in the case of SR1 and SR2 are shown in Figure 6. It can be seen that in the case of SR1, all three algorithms can reconstruct good HRRPs. However, at SR2, due to the influence of high grating and side lobes, severe errors occur in the HRRPs reconstructed by S-CLEAN. Figure 7 shows the micro-motion curves reconstructed by the three algorithms corresponding to precession at the two SRs. Since the S-CLEAN method is used to extract the maximum peak point to achieve the suppression of high grating and side lobes, with the decrease in SR, the error arising from extracting peak point leads to the micro-motion curve reconstruction failure. 2D-CPA fails to make full use of 2-D coupling information and 2-D coherent accumulation gain of echo signal due to cascaded processing, and is prone to the estimation error transmission, so its reconstruction accuracy is insufficient. In contrast, 2D-JSR-MPE can accurately reconstruct the micro-motion curve in the same condition.

**Figure 8:**
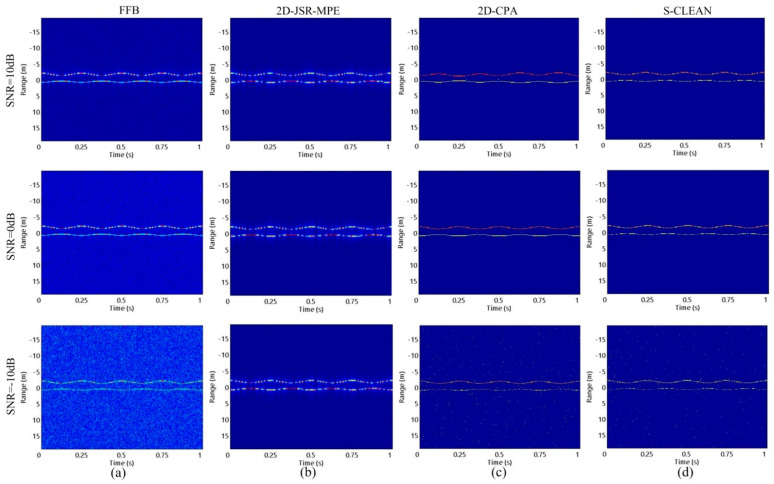
The reconstructed results of HRRP of the target with precession motion obtained by different algorithms with different SNRs at SR = 0.75: (**a**) FFB situation; (**b**) 2D-JSR-MPE; (**c**) 2D-CPA; (**d**) S-CLEAN.

**Figure 9:**
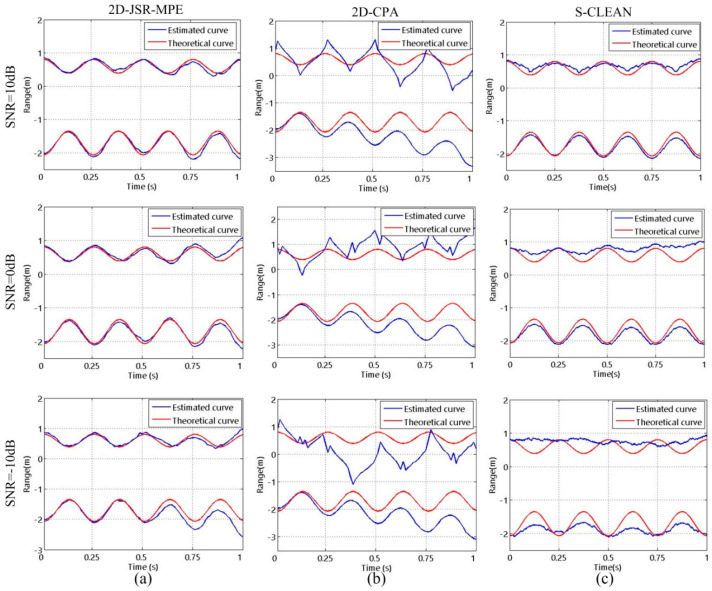
The reconstructed results of micro-motion curves of the target with precession motion obtained by different algorithms with different SNRs at SR = 0.75: (**a**) 2D-JSR-MPE; (**b**) 2D-CPA; (**c**) S-CLEAN.

In order to study the influence of SNR on the algorithms, HRRPs are reconstructed by 2D-JSR-MPE, 2D-CPA and S-CLEAN at SNR = −10 dB, 0 dB and 10 dB. The homologous results are shown in Figure 8. It can be seen that there are a lot of false points in the HRRPs reconstructed by 2D-CPA and S-CLEAN at low SNR, but 2D-JSR-MPE succeed in reconstructing HRRPs accurately. Figure 9 reveals the micro-motion curves reconstructed by the three algorithms under the three SNR conditions. As SNR decreases, 2D-CPA and S-CLEAN are seriously disturbed by noise, and the error of the reconstructed micro-motion curves increases gradually. However, compared with the above two comparison algorithms, 2D-JSR-MPE can still reconstruct the accurate micro-motion curves at different SNRs. The experiments above corroborate the good robustness of the proposed algorithm against SR and SNR.

**Figure 10:**
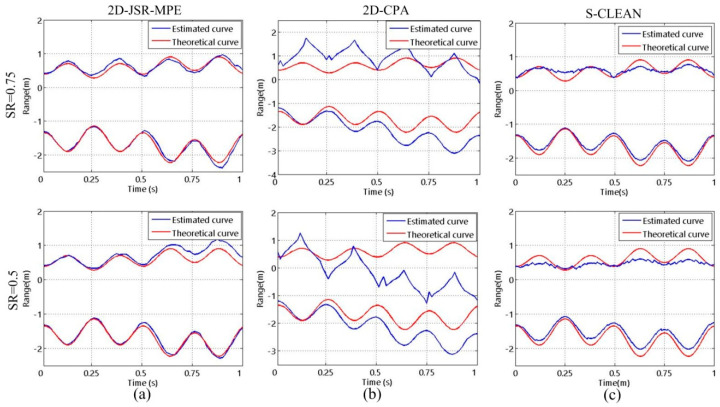
The reconstructed results of micro-motion curves of the target with nutation motion obtained by different algorithms with different SRs at SNR = 10 dB: (**a**) 2D-JSR-MPE; (**b**) 2D-CPA; (**c**) S-CLEAN.

**Figure 11:**
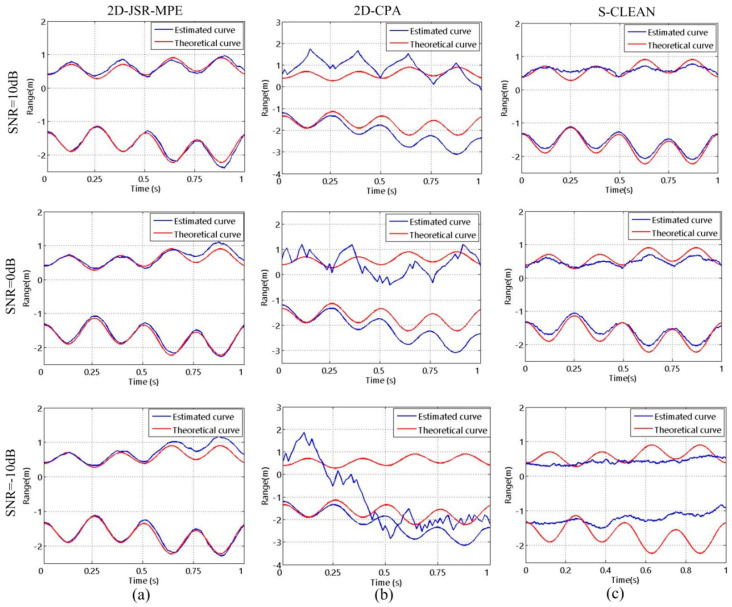
The reconstructed results of micro-motion curves of the target with nutation motion obtained by different algorithms with different SNRs at SR = 0.75: (**a**) 2D-JSR-MPE; (**b**) 2D-CPA;(**c**) S-CLEAN.

In addition, based on the precession motion of the target, the oscillating motion of the symmetry axis of the target is introduced into the experiment to estimate the micro-motion curves of the target with nutation motion. Figure 10 and Figure 11 are the corresponding micro-motion curves reconstructed by the three algorithms at the two kinds of SRs and the three types of SNRs when the target is in nutation. They suggest that the experimental conclusions drawn in the complex nutation are the same as those acquired in the precession, which further verify the effectiveness of the proposed algorithm.

**Figure 12:**
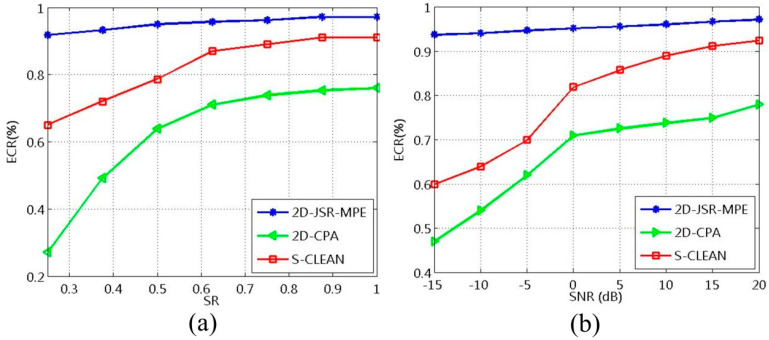
ECRs obtained by different algorithms: (**a**) Different algorithms with different SRs at SNR = 10 dB; (**b**) Different algorithms with different SNRs at SR = 0.75.

For the purpose of analyzing the impact of SR and SNR on the performances of the proposed algorithm and the two comparison algorithms quantitatively, the estimated correct rate (ECR) of the reconstructed micro-motion curve is defined by (20)

(20) $$ECR=\left(1-\frac{\sum_{t_{m}}\left|F_{r}\left(t_{m}\right)-F_{e}\left(t_{m}\right)\right|}{\sum_{t_{m}}\left|F_{r}\left(t_{m}\right)\right|}\right)*100\%$$

where $F_{e}\left(t_{m}\right)$ represents the estimated value of the micro-motion curve and $F_{r}\left(t_{m}\right)$ the true value of the micro-motion curve. Firstly, the influence of SR on the performance of the algorithms is analyzed. In the case of precession motion, with other conditions unchanged, SR ranges from 0.25 to 1 in step of 0.125 at SNR = 10 dB. Without loss of generality, 40 groups of echo data are collected at each SR, followed by the reconstruction of their micro-motion curves. After calculating the ECR of each curve, the average value of 40 groups of ECRs can be obtained, which is shown in Figure 12a. Compared with the other two algorithms, the proposed one has higher ECRs at all SRs.

Finally comes the analysis of the influence of SNR on the algorithms’ performance. Assuming other conditions remain unchanged, the robustness of the algorithms against noise is analyzed with SR = 0.75 and SNR ranging from −15 to 20 dB in step of 5 dB. The average ECR is calculated by the same method, and the result is shown in Figure 12b. It can be seen that the ECRs of the proposed algorithm are above 90% at different SNRs, higher than those of the other two algorithms. This serves as convincing proof of the good robustness of the proposed algorithm against noise.

## 5. Conclusions

This paper proposes a novel 2D-JSR-MPE algorithm. Through piecewise processing, we establish the 2-D joint sparse reconstruction signal model and the target’s micro-motion characteristic parameter dictionary, in which the idea of piecewise effectively reduces the model complexity of ballistic target. Based on the CS theory, an improved OMP algorithm is employed to solve a sparse optimization problem with $l_{1}$-norm. With the help of 2-D joint processing, the precise reconstruction of HRRP and micro-motion curve can be realized simultaneously, thereby avoiding the error transmission between HRRP reconstruction and micro-motion parameters estimation. Making full use of the 2-D coupling information and 2-D coherent accumulation gain of echo signal, the proposed algorithm can accurately reconstruct the micro-motion curve of the ballistic target on phase accuracy. Extensive experimental results demonstrate that the proposed algorithm can be implemented with high accuracy and strong robustness at different SNRs and SRs. Further efforts to improve the performance of our work in this paper in the case of sparse frequency band and sparse aperture (SFB-SA) echo signal of ballistic target are underway.

## Figures and Tables

**Table 1:** Main simulated parameters of the ballistic target.

Parameters	Values
Height of the target	4.0 m
Distance between the mass center and the top of the target	2.7 m
Radius of the bottom	0.3 m
Spinning frequency	2 Hz
Coning frequency	4 Hz
Oscillating frequency	1 Hz
Precession angle	10°

**Table 2:** Main simulated parameters of the radar system.

Parameters	Values
Carrier frequency	10 GHz
Bandwidth	1 GHz
Pulse repetition frequency	4 kHz
Coherent processing interval	1 s
